# Integrated Microfluidic Flow-Through Microbial Fuel Cells

**DOI:** 10.1038/srep41208

**Published:** 2017-01-25

**Authors:** Huawei Jiang, Md. Azahar Ali, Zhen Xu, Larry J. Halverson, Liang Dong

**Affiliations:** 1 Department of Electrical and Computer Engineering, Iowa State University, Ames, IA 50011, USA; 2 Department of Plant Pathology and Microbiology, Iowa State University, Ames, IA 50011, USA

## Abstract

This paper reports on a miniaturized microbial fuel cell with a microfluidic flow-through configuration: a porous anolyte chamber is formed by filling a microfluidic chamber with three-dimensional graphene foam as anode, allowing nutritional medium to flow through the chamber to intimately interact with the colonized microbes on the scaffolds of the anode. No nutritional media flow over the anode. This allows sustaining high levels of nutrient utilization, minimizing consumption of nutritional substrates, and reducing response time of electricity generation owing to fast mass transport through pressure-driven flow and rapid diffusion of nutrients within the anode. The device provides a volume power density of 745 μW/cm^3^ and a surface power density of 89.4 μW/cm^2^ using *Shewanella oneidensis* as a model biocatalyst without any optimization of bacterial culture. The medium consumption and the response time of the flow-through device are reduced by 16.4 times and 4.2 times, respectively, compared to the non-flow-through counterpart with its freeway space volume six times the volume of graphene foam anode. The graphene foam enabled microfluidic flow-through approach will allow efficient microbial conversion of carbon-containing bioconvertible substrates to electricity with smaller space, less medium consumption, and shorter start-up time.

Microbial fuel cells (MFCs) utilize bacteria as a biocatalyst to oxidize organic matter and release electrons that can be harvested to generate electricity^1^. Because MFCs can remove organic matter from wastewater and simultaneously produce renewable energy, the use of MFCs to achieve sustainable wastewater treatment is an attractive alternative to traditional treatment processes^2^. In addition, MFCs have been suggested as an in-field energy source to power microscale sensors for agricultural, environmental, and process monitoring^3^,^4^,^5^,^6^,^7^,^8^,^9^,^10^,^11^,^12^. However, currently, the main applications of MFCs remain confined to laboratory-scale devices. A limiting factor for the progress of using MFCs for field applications is their limited power density^2^. Therefore, there is a concerted worldwide effort to advance MFC technology and maximize their translational potential toward large-scale practical applications^1^,^2^,^3^.

Miniaturized MFC (μMFC) technologies have received increased attention, owing to their great potential to realize high-throughput screening of different bacterial strains for high-efficiency conversion of substrates to electricity^2^,^13^,^14^,^15^,^16^,^17^,^18^,^19^,^20^,^21^,^22^,^23^,^24^,^25^,^26^,^27^. Generally, μMFCs are featured by low material consumption, short start-up time, easy operation, and experiment parallelization^13^. To improve current and power densities of μMFCs, researchers have made significant progress in optimizing bacterial strains^16^,^17^,^21^,^22^, device structures^18^,^19^,^20^,^24^,^25^,^26^, and anode materials^23^,^24^,^25^,^26^,^27^,^28^,^29^,^30^,^31^,^32^,^33^,^34^,^35^,^36^,^37^,^38^,^39^,^40^,^41^,^42^,^43^,^44^,^45^,^46^. For example, due to the large surface area-to-volume ratio, many micro/nanomaterials have been developed as anode materials of μMFCs to promote bacterial attachment and colonization, and electrochemical catalytic activity of anodes, such as carbon nanotubes (CNTs)^23^,^28^,^43^,^44^, graphene^45^, graphene-based nanocomposites^27^,^29^,^30^, poly(3,4-ethylenedioxy-thiophene) (PEDOT)^31^,^32^,^46^, and PEDOT-based nanocomposites^34^,^35^,^36^,^37^,^38^,^39^,^40^,^41^,^42^. Despite these efforts, it remains challenging in obtaining high current and power intensities for μMFCs, due to their small processing volume and insufficient biofilm formation. Interestingly, most existing μMFCs employ a similar device structure where carbon-containing organic substrate solutions flow over the surface of a planar metal anode (e.g. gold) or micro/nanomaterials-based anode emplaced on the bottom of anolyte chamber or attached to a proton exchange membrane (PEM). During the batch mode operation, mass transport of nutrients to the microbes colonizing the anode surface is often implemented through a relatively slow diffusion process from the bulk solution outside the anode to the surface or inside of the anode. In the continuous flow mode, some of the bioconvertible substrates are wasted as they directly flow out from the anolyte chamber through the freeway space outside the anode, without contributing to the electricity generating biofilm-mediated metabolic reactions occurring at the anode surface.

With continuing efforts in miniaturizing MFCs, the volume of substrate solutions decreases dramatically, thus necessitating increasing the efficiency by which the nutrients are made available to microbes colonizing the anode. Notably, microchannelled nanocomposites made of CNTs and chitosan were recently developed as acetate-oxidizing bioanodes in relatively large bioelectrochemical devices. The electrode allowed flowing of bacterial culture through the nanocomposite anode to promote the growth of electroactive bacterial films^47^. But, it is unclear how such engineered device structure can affect nutrient utilization and mass transport of nutrients inside the device. Elucidating and understanding the nature of these variables are of crucial importance for designing high-performance μMFCs to sustain electron production of the biofilm colonized on the anode surface. Interestingly, a microfluidic vanadium redox fuel cell was reported utilizing carbon paper based electrodes to enable cross-flow of the fuel and oxidant solutions through the electrodes into an exit channel^48^. This remarkable architecture increased the active area of vanadium redox reactions and enhanced rates of mass transport inside the anode. However, to our knowledge, flow-through (FT) transport mechanism has not been reported for integrated and miniaturized fuel cells using microbes as biocatalysts.

In this paper, we report an integrated microfluidic FT μMFC using 3D graphene foam (GF) as anode able to minimize bioconvertible substrate consumption, sustain high levels of nutrient utilization, and reduce response time of electricity generation (Fig. 1). GF is a porous conductive structure formed by vapor deposition of graphene onto a 3D mesh of metal filaments. It has been utilized in electronic devices^27^,^28^,^29^, energy storage and conversion devices^32^,^33^,^34^,^35^,^36^,^37^,^38^,^39^,^40^,^41^,^42^, and neural tissue engineering^49^,^50^,^51^. Recently, GF, with the pore size of a few hundred micrometers, has also been employed as anode material of MFCs and demonstrated that the scaffold of GF is favorable for bacteria colonization and electron transfer^27^,^29^,^30^,^52^. It should be noted that all the existing GF-based MFCs adopted a traditional non-FT design where the nutritional media flow over the anode. In the proposed microfluidic FT device, the porous GF anode is seamlessly embedded in the anolyte chamber and directly sandwiched between a PEM and a gold electron collector at the bottom of the anolyte chamber. No nutritional media flow over the anode. With the built-in interconnected pore network, the embedded GF anode can not only provide 3D scaffolds for bacterial attachment and colonization, but more importantly serve as a natural microfluidic porous channel for flowing nutrient solutions over the biofilm formed on the scaffolds. The catholyte chamber located on the other side of the PEM contains carbon cloth, which acts as the cathode. The porous GF anode provides numerous passages for nutritional media to flow through the anode. By minimizing the freeway space outside the anode, the waste of nutritional media through flowing over the anode without interacting with the bacteria colonizing the porous scaffold anode will be avoided or greatly minimized. Here, mass transport is driven by pressure and diffusion of nutrients directly inside the interstitial pores of the GF. Over the micrometer length scale, molecular diffusion is fast because the diffusion time scales as the square of the distance. Therefore, this present microfluidic FT strategy provides increased transport of bioconvertible substrates from the bulk to the active area inside the GF.

## Experimental

### Chemicals and materials

The following materials were used for fabrication of the proposed μMFCs: GF (multilayer freestanding graphene foam; area: 7 × 7 mm^2^; thickness: 1.2 mm; mean pore size: 580 μm; density: 4 mg/cm^3^; Graphene Supermarket, Calverton, NY), PEM (Nafion 117; Fuel Cells, College Station, TX), carbon cloth (Fuel Cell Store, College Station, TX), acrylic sheets (85 mm × 35 mm × 3 mm; TAP Plastics, Oakland, CA), glass slides (75 mm × 25 mm × 0.9 mm, Corning, Oneonta, New York), a photopolymerizable precursor solution for making microfluidic channels composed of isobornyl acrylate (IBA, Sigma-Aldrich, St. Louis, MO), tetraethylene glycol dimethacrylate (Sigma-Aldrich, St. Louis, MO), and 2,2-dimethoxy-2-phenylacet ophenone (Sigma-Aldrich, St. Louis, MO) with a weight ratio of 32:1.7:1.0, polyethylene sterile tubing (Cole-parmer, Vernon Hills, IL), and mechanical cap screws (M4 × 0.7; Thorlabs, Newton, NJ). Here, the PEM was pretreated by sequentially boiling in a 30% hydrogen peroxide solution in water (Fisher Scientific, Fair Lawn, NJ), followed by soaking in a 0.5 M sulfuric acid solution (Fisher Scientific, Fair Lawn, NJ) and then DI water, each for hour^18^. The activated PEM was stored in DI water before assembly.

Tryptic soy broth (TSB, Sigma-Aldrich, St. Louis, MO) was used as a culture medium for *S. oneidensis* strain MR-1. A lactate defined minimal medium used for electrochemical measurements was comprised of the following: 20 mM sodium lactate per liter of DI water, 28 mM NH_4_Cl, 1.34 mM KCl, 5 mM NaH_2_PO_4_, 0.7 mM Na_2_SO_4_, 52 mM NaCl, 0.2 mM CaCl_2_ (the above chemicals purchase from Fisher Scientific, Fair Lawn, NJ), 1 mM MgSO_4_·7H_2_O, 10 mg FeSO_4_·7H_2_O (the two chemicals purchase from Alfa Aesar, Ward Hill, MA), 20 mM PIPES [piperazine-*N,N*’-bis (2-ethanesulfonic acid)] (Sigma-Aldrich, St. Louis, MO), and 1 mL trace element solution^53^. Potassium ferricyanide (Fisher Scientific, Fair Lawn, NJ) was used as catholyte solutions. For studying electrochemical activity of GF anode electrode, the phosphate buffered saline (PBS) containing 5 mM [Fe(CN)_6_]^3−/4−^ (pH = 7.0) was used, where [Fe(CN)_6_]^3−/4−^ acted as a redox mediator.

### Device fabrication and assembly

**Figure 2:**
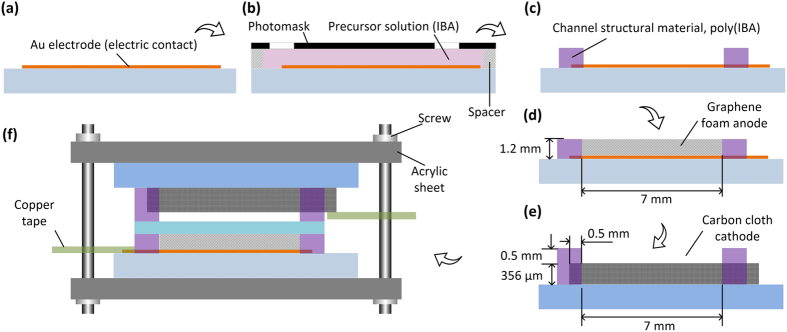
Fabrication processes for a microfluidic FT μMFC.

The gold electron collector (100-nm-thick Ti/Au) was first formed on the glass slide by e-beam evaporation of gold and conventional photolithography with the help of a film photomask (Fineline Imaging, Colorado Springs, CO) (Fig. 2a). Subsequently, the anolyte chamber (area: 7 × 7 mm^2^; depth: 1.2 mm) was fabricated on the same glass slide using a liquid phase polymerization process (LP^3^)^54^. The diverging channels were designed on the two sides of the porous anolyte chamber to distribute the anolyte solution flow relatively evenly across the width of the anolyte chamber. In the LP^3^ step, double sided tapes (3 M, St. Paul, MN) were used as spacers to create a 1.2 mm deep cavity between the glass slide and another film photomask (Fig. 2b). The IBA-based photosensitive precursor solution was then injected into the cavity and polymerized under ultraviolet light (8.4 mW/cm^2^; 30 s)^54^, after which the glass slide was washed with pure ethanol and then baked at 60 ^◦^C for 2 hrs (Fig. 2c). A strip of carbon cloth (area: 6 mm × 15 mm; thickness: 356 μm) was placed on another glass slide, followed by the LP^3^ process to form the catholyte chamber. As shown in Fig. 2e, three sides of the carbon cloth strip overlapped the photomask by 0.5 mm to fix the carbon cloth to the bottom of the catholyte chamber. Finally, the device was constructed by assembling all the components (Fig. 2f). These components were clamped between two acrylic plates and held together by four cap screws. To build the electrical connections, copper tapes (Sparkfun, Niwot, CO) were used to extend the anode and cathode. The anolyte and catholyte chambers were accessed by sterile polyethylene tubing through inlet and outlet ports.

### Cell inoculation

The μMFCs were sterilized by filling all of compartments with a 70% ethanol in water and letting it sit for 20 min at room temperature, followed by flushing the device with sterile DI water for 5 min prior to TSB culture medium for 5 min. To operate the device, *S. oneidensis* strain MR-1 was used as the model exoelectrogenic microbial biocatalyst and TSB was used the nutrient source. TSB medium flowed into the anolyte chamber through the polyethylene tubing using a programmable syringe pump (210P, KD Scientific, Holliston, MA). To minimize possible oxygen contamination in the batch mode operation, the tubing was closed by steel clamps after the injection of bacterial suspension into the anolyte chamber. The catholyte solution of potassium ferricyanide was supplied using a syringe pump.

### Measurement and calculation

An external resistor (*R*) was connected between the anode and cathode to form a closed circuit. The voltage potential (*U*) between the two electrodes was measured using a data acquisition device (Model DI-245; DATAQ Instruments, Akron, OH) and recorded once a minute via DATAQ Instruments Hardware Manager software. The current (*I*) flowing through the resister was calculated *via I* = *U*/*R* and the output power was calculated *via P* = *U* × *I*. The shunt current was measured to obtain the maximum output current. Electrochemical properties of the GF anode was measured in PBS containing 5 mM [Fe(CN)_6_]^3−/4−^ by an electrochemical workstation (SP1, Zive Potentiostat, Seoul, Korea). A platinum (Pt) wire and a silver/silver chloride (Ag/AgCl) wire were used as the counter and reference electrodes, respectively. Each electrical measurement result given in this paper is representative of the typical result obtained over three independent experiments on three devices.

Coulombic efficiency (*CE*) of μMFCs operated in batch mode was calculated as *CE* = (*C*_P_/*C*_T_) × 100%, where *C*_P_ is the total coulombs generated by the device within one batch, and *C*_T_ is the total amount of coulombs theoretically available. Specifically, *C*_P_ was obtained by integrating the total area in the current (*I) versus* time (*t*) plot during lactate consumption and described as 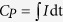. *C*_T_ was calculated as *C*_T_ = *n* × *F* × *V* × [lactate], where *n* is the electron transfer constant of the substrate representing the mole of electrons yielded by oxidation of 1 mol lactate into 1 mol acetate at the anode, *F* is Faraday’s constant, *V* is the volume of anode chamber, and [lactate] is the initial concentration of sodium lactate in the lactate defined minimal medium (here, 20 mM). The electrochemical reaction occurred at the anode and cathode of the device are listed as follows:


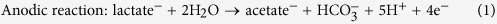



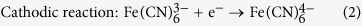


### Bacterial fixation for SEM

The GF anodes were separated from the disassembled μMFCs, immersed in a glutaraldehyde solution (2%; Sigma-Aldrich, St. Louis, MO) to fix the adherent bacteria on the GF surface, and incubated at 4 ^◦^C for 12 hrs. After rinsing with water, the GF anodes were stained with 1% osmium tetroxide solution (Sigma-Aldrich, St. Louis, MO) for 2 hrs, rinsed again, and then, dehydrated with pure ethanol. A field-emission scanning electron microscope (SEM; Quanta-250; FEI, Hillsboro, OR) was used.

## Results and Discussion

**Figure 3:**
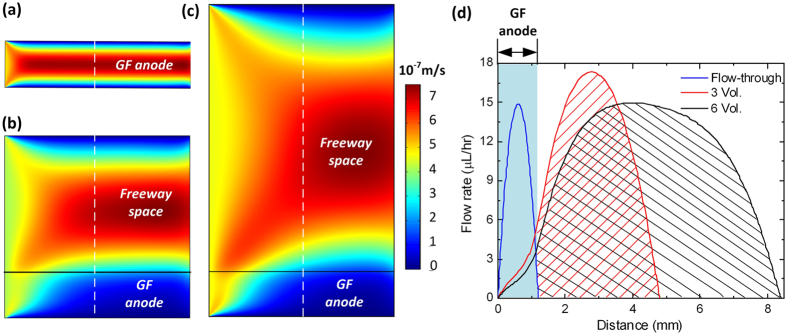
Simulated flow rate distributions over the vertical cross-section of the anolyte chambers of three sample μMFCs: a FT (**a**), and two non-FT devices with the freeway space height (above the GF anode) three (**b**) and six times (**c**) the thickness of the GF anode. In (**d**), simulated flow rates along the dashed white lines across the width of the anolyate chambers are given.

To illustrate how the microfluidic FT design affected fluid flow in the anolyte chamber, we conducted hydrodynamic simulation using a finite element method based commercial software package (COMSOL Multiphysics). Figure 3 shows simulated flow rate distributions of the FT design with the GF anode sandwiched by the PEM and the glass slide, and two non-FT counterparts with freeway space height of three and six times the thickness of the GF anode, respectively. Free- and porous-media flow models were used for the simulations. For the porous media, the porosity, permeability, and Forchheimer coefficient were set as 0.9, 1 × 10^−7^ m^2^, and 4.35 × 10^3^ kg/m^4^, respectively^55^. In the settings for fluid properties, the density, dynamic viscosity, and flow rate were set as 1 × 10^3^ kg/m^3^, 1.02 × 10^−3^ Pa·s, and 4.6 × 10^−4^ cm/s (calculated by a sample volumetric flow rate of 10 μL/hr used in the experiment), respectively. The width and side length of the porous media were set to 1.2 mm and 5 mm, respectively, according to the geometric parameters used in the real device.

Figure 3a shows that as the pressure driven laminar flow was pumped through the FT porous anolyte chamber, the fluid velocity approaches zero the closer to the walls generating a parabolic velocity profile within the chamber in each case. The parabolic velocity profile has significant implications for the distribution of molecules transported within the anolyte chamber. Specifically, in the microfluidic FT device all fluids would travel through the porous anode, while in the two non-FT devices, only a portion of the fluids would directly interacted with the GF anode, with a majority of the fluids flowing through the freeway space channel as waste. The wider the freeway space, the lower the flow rate inside the porous anode and the lower the efficiency use of the medium. Importantly, the microfluidic FT design will allow for faster nutrient replenishment due to the presence of a higher flow rate inside the GF anode and less waste of nutrients.

Electric current output of the microfluidic FT device before and during cell inoculation was monitored using a closed circuit carrying an external resistive load of 11.5 kΩ. When TSB culture medium was injected into the anolyte chamber the background current without *S. oneidensis* bacteria was as low as 12 ± 10 nA. When TSB with *S. oneidensis* cell suspensions was injected into the anolyte chamber the output current increased to ~43 μA within five hours and then decreased gradually over time. The magnitude of peak current was three orders more than the background current. Considering that the inoculum was not manipulated prior to delivering it to the anolyte, the rapid increase in electric current output is due to the rapid metabolism of nutrients by the electrogenic bacteria. The decrease in output over time is likely the consequence of nutrient depletion in the batch mode.

**Figure 4:**
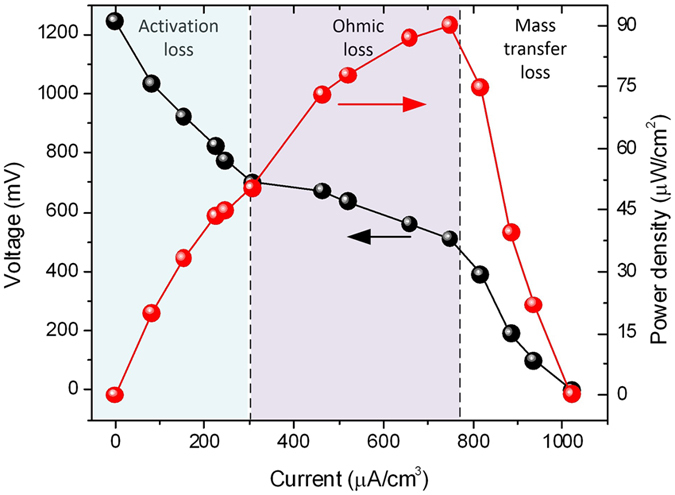
Polarization curve (black symbols) and power density output (red symbols) curves of the FT μMFC as a function of current density.

Figure 4 shows the polarization and power density curves of the microfluidic FT device plotted by measuring the output voltage and current density at different external resistive loads. According to the literature^1^, a polarization curve can be divided into three regions reflecting activation loss, ohmic loss, and mass transfer loss. At the activation loss stage, the output current density increased from zero (measured at the open circuit voltage of 1240 mV) to 300 μA/cm^3^. The voltage dropped as the external resistance decreased. In the current range of 300 to 765 μA/cm^3^, there was a near-linear drop in voltage with increasing electric current. As a result, an internal resistance was estimated to be 7.3 kΩ through linear fitting of the curve in the ohmic loss region. In addition, the surface power density reached a maximum of 89.8 μW cm^−2^ in the ohmic loss region, while the output voltage decreased in the mass transfer loss region.

**Figure 5:**
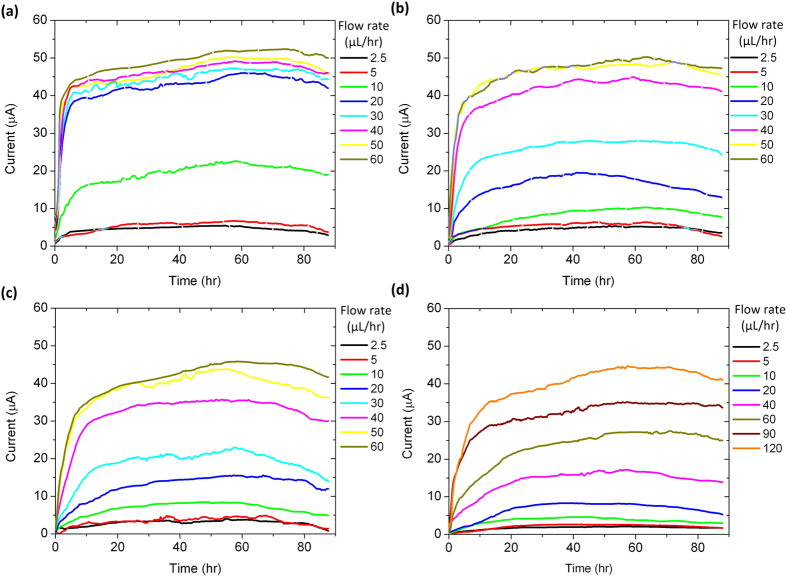
Typical output current of a microfluidic FT device (**a**) and three non-FT devices with the freeway space volume *V*_fr_ = *V*_gf_ (**b**), 3*V*_gf_ (**c**), and 6*V*_gf_ (**d**), in response to feeding TSB medium at different TSB medium flow rates.

To evaluate the influence of the feeding rate of culture medium on the electric current generation of the microfluidic FT device, TSB medium was continuously injected into the FT anode at different flow rates after inoculating the chamber with *S. onediensis*. As shown in Fig. 5a, the output currents at 2.5 and 5 μL/hr were similar (~5 μA) to each other. But, with increasing flow rates at or above 10 μL/hr, there were dramatic current increases. It is interesting that the electric currents at flow rates greater than 20 μL/hr were almost saturated at the level of 40–50 μA, with only a small current increase as the flow rate increased. A possible explanation is that the nutritional supply to microbes colonizing the GF surfaces at the flow rate of 20 μL/hr may be sufficient to support near maximal cell growth and respiration. Therefore, further increasing the feeding flow rate beyond 20 μL/hr had only limited influence on electricity production.

**Figure 6:**
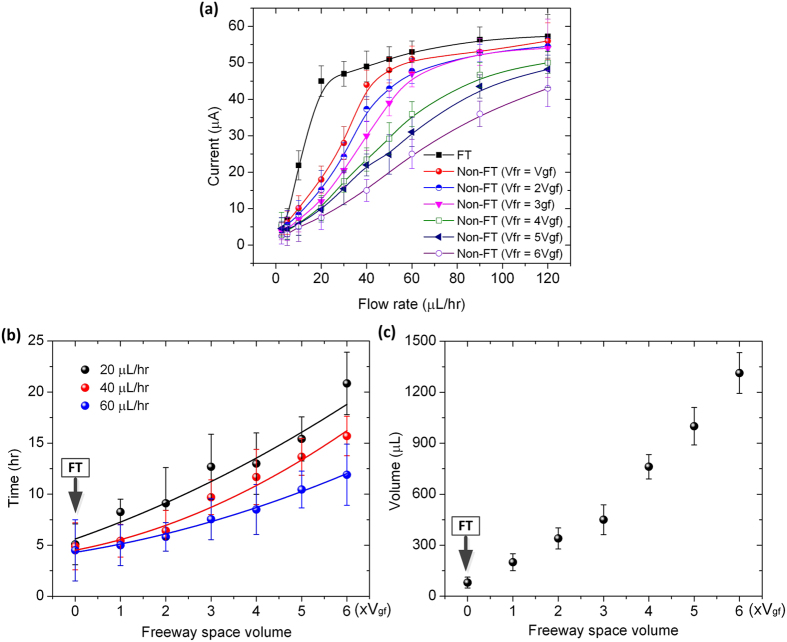
(**a**) Maximum output current of the microfluidic FT and non-FT devices at different TSB medium flow rates. The non-FT devices used here have the freeway space volume *V*_fr_ varying from *V*_gf_ to 6*V*_gf_ (*V*_gf_ represents the volume of GF anode). (**b**) Time required for the FT and non-FT devices to obtain 80% of the peak output current as a function of freeway space volume of the devices. (**c**) Total volume of TSB medium consumed to obtain 80% of the peak output current as a function of freeway space volume of the devices.

To reveal the advantages of the microfluidic FT device, we fabricated different non-FT counterpart devices with the freeway space volume (*V*_fr_) above the GF anode varied from one to six times the GF anode volume (*V*_gf_). Cell inoculation for the non-FT devices was performed with the same procedures as those performed for the microfluidic FT device described above. After the completion of inoculation, we tested electric current generation of the non-FT devices at different medium flow rates in a continuous flow mode. The representative results of three non-FT devices (*V*_fr_ = *V*_gf_, 3*V*_gf_, and 6*V*_gf_) are shown in Fig. 5b–d, where the overall tendencies of electric current output over time at different flow rates are similar to that of the FT device (Fig. 5a), but greater flow rates were required to achieve comparable current outputs. For example, in order to produce a 35 μA current, the non-FT devices with *V*_fr_ = *V*_gf_, 3*V*_gf_, and 6*V*_gf_ required a continuous supply of nutritional medium at a flow rate of 40 μL/hr for ~6 hrs (Fig. 5b), at 60 μL/hr for ~9 hrs (Fig. 5c), and at 120 μL/hr for ~12 hrs (Fig. 5d), respectively. In contrast, for the microfluidic FT device, the same current was produced with a much lower flow rate of 20 μL/hr for a shorter period of ~5 hrs (Fig. 5a). This is because the microfluidic FT device could deliver more nutrients to the bacteria colonizing the graphene foam, whereas in those non-FT devices, only a fraction of the input resources were delivered to the microbes inside the GF anode. Figure 6a summaries the output current of the microfluidic FT and non-FT devices as a function of medium flow rate.

Figure 6b shows the time (*t*_I_) that the microfluidic FT device and six non-FT counterparts took to generate 80% of their corresponding peak current with respective flow rates of 20, 40, and 60 μL/hr. The result shows that the FT design allowed for significant reduction in *t*_I_. The larger the freeway space volume of the non-FT device, the longer the time *t*_I_ of the device. Along the length direction of the GF anode in the FT device, mass transport of nutrients was mainly driven by pressure in the continuous flow mode. As a result, the higher the feeding flow rate the shorter the time *t*_I_ required. Inside the interstitial pores of the GF anode, nutrients could diffuse to the surface of scaffolds over a short length of approximately 290 μm or half of the mean pore size of the GF (mean pore size: 580 μm; see Section Chemicals and materials), which also contributed to the short *t*_I_ of the FT device. In contrast, the non-FT devices required more time because the nutrients in the freeway space outside the anode diffused over a longer distance to the scaffolds of the GF anode. Although pressure-driven mass flow also occurred in the non-FT devices, the effective amount of nutrients delivered to the colonized microbes was actually less than that in the FT device. As shown in Fig. 6b, when the medium flow rate was set at 20, 40, and 60 μL/hr, the response time *t*_I_ of the FT device was 4.2, 3.2, and 2.6 times, respectively, shorter than that of the non-FT device with *V*_fr_ = 6*V*_gf_.

**Figure 7:**
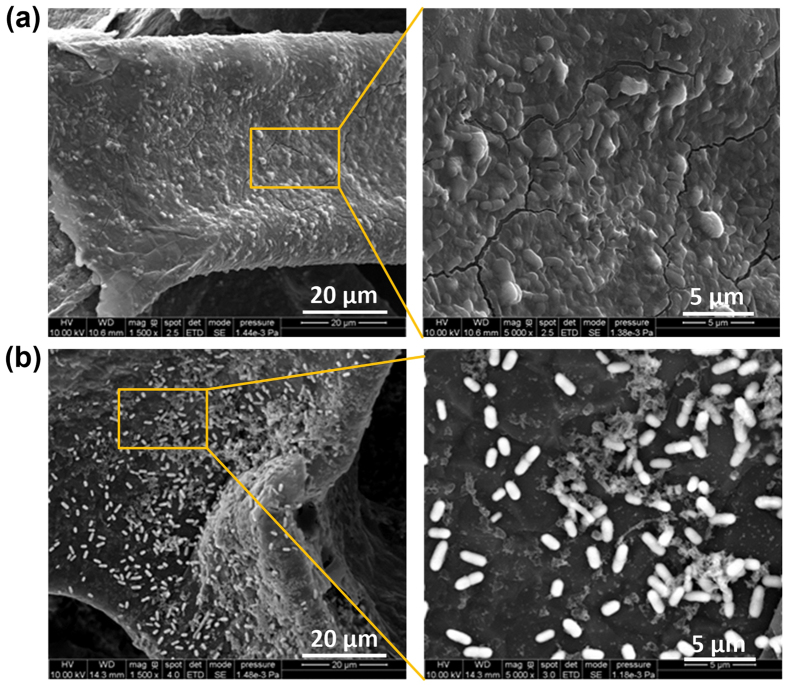
SEM images for the biofilms of *S. oneidensis* strain MR-1 grown on the scaffolds of the GF anodes of the FT device (**a**) and non-FT counterpart with V_fr_ = 6V_gf_ (**b**). The biofilms were formed at TSB medium flow rate of 20 μL/hr. The incubation time was 80 hours in each device.

Figure 7a,b show the biofilms of *S. oneidensis* strain MR-1 formed on the scaffolds of the GF anode taken out from the microfluidic FT device and the non-FT counterpart with *V*_fr_ = 6*V*_gf_, respectively. The biofilms were examined following the continuous-flow mode operation at the same flow rate of 20 μL/hr. As described in Figs 5a,d and 6a, at this flow rate the FT device generated the electric current (~45 μA) about 7.5 times that generated by the non-FT device (~6 μA). The SEM images shows that the surfaces of scaffolds in the FT device was fully covered by the biofilm of *S. oneidensis* strain MR-1, while those in the non-FT device were only partially covered by the bacteria.

To better understand the operation of the GF anode in the microfluidic FT and non-FT μMFC devices, we investigated electrochemical properties of the GF anode by cyclic voltammetry (CV) and electrochemical impedance spectroscopy (EIS). This helped us to develop some insight into how diffusion coefficients of electroactive species were related to the μMFC design with the GF as an anode. It should be noted that because TSB medium is a chemically complex medium, the diffusion coefficient obtained through electrochemical measurements only reflect the overall ability of diffusing molecules in the anolyte chamber, but not any individual molecules. Therefore, we also used a lactate defined minimal medium for *S. onediensis* MR-1 to perform electrochemical measurements for the FT device and the non-FT counterpart (V_fr_ = 6 V_gf_).

**Figure 8:**
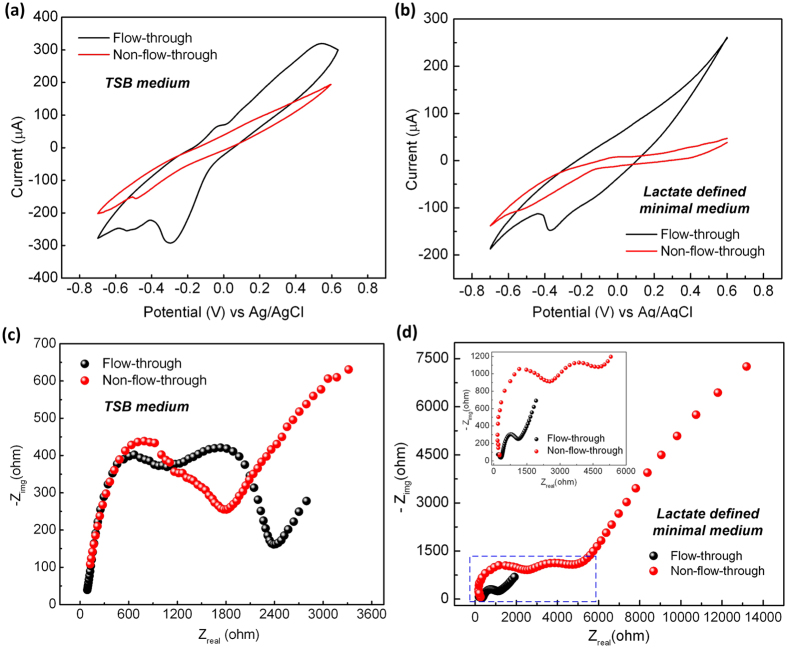
Overlay of CV characteristic curves of the GF anodes in the microfluidic FT and non-FT μMFCs at the scan rate of 30 mV/s with TSB medium (**a**) and lactate defined minimal medium (**b**). Nyquist plots of the GF anodes for the microfluidic FT and non-FT devices with TSB medium (**c**) and lactate defined minimal medium (**d**) in EIS studies. The inset in (**d**) shows the enlarged view of the indicated square region in the same figure.

The CV studies were carried out in both TSB and the lactate defined minimal medium at 30 mV/s scan rate within a potential range of 0.6 V to −0.7 V for the GF anode and the carbon cloth cathode. The reduction current with TSB medium was higher in the microfluidic FT device (−292 μA at −0.3 V) than in the non-FT device (Fig. 8a). The higher current indicates faster electron transfer to the anode. According to Bard and Faulkner^56^, when diffusion process dominates in the electrochemical reactor, the peak current can be given by 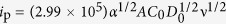, where *α* is the transfer coefficient for the reaction; *A* is the surface area of the anode (cm^2^), ν is the scan rate (mV/s), *C*_o_ is the initial concentration of substrate in the medium (mol/cm^3^), and *D*_0_ is the diffusion coefficient (cm^2^/s). In the case of TSB medium (Fig. 8a), the average diffusion coefficients were 2.38 × 10^−10^ and 0.2 × 10^−10^ cm^2^/s for the microfluidic FT and non-FT device, respectively (Table 1). The higher average diffusion coefficient of the FT device is presumably due to the shorter diffusion path. Similarly, due to the same reason, in the case of the lactate defined minimal medium (Fig. 8c), the FT device provided a higher reduction current of −148 μA with a larger diffusion coefficient of 6.28 × 10^−9^ cm^2^/s than the non-FT one (1.40×10^−9^ cm^2^/s).

Furthermore, we compared other kinetic parameters of the microfluidic FT and non-FT devices (Table 1), including charge transfer resistance (*R*_ct_), and heterogeneous electron transfer rate constant (*k*_o_), based on the results of EIS measurements by applying a small (amplitude: 10 mV) sinusoidal AC signal (frequency range: 0.01 Hz to 100 *k*Hz). In EIS, a Nyquist plot includes a semicircle region with the real axis indicating *R*_ct_ (plotted with the real part *Z*_real_ and the imaginary part *Z*_img_). The *k*_0_ of the μMFC was calculated using the following equation: 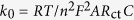, where *T* is the temperature, *R* is the gas constant, *n* is the electron transfer constant of the substrate (for TSB medium, *n* = 24; for the lactate defined minimal medium, *n* = 4), *A* is the anode area, and *C* is the concentration of the substrate. Figure 8c,d show that the EIS spectra for the two μMFC designs using TSB and lactate based culture media, respectively. In the case of the FT device with TSB medium, the first semicircle appeared in the Nyquist plot with *R*_ct1_ = 1.23 kΩ at high frequencies, respectively. At high frequencies with TSB medium, the non-FT device had a charge transfer resistance *R*_ct_ of 1.44 kΩ, which is higher than that the FT device provided. Also, the microfluidic FT device had the higher *k*_0_ values with both TSB and lactate defined minimal media, compared to the non-FT one. Therefore, the FT device exhibited faster electron transfer kinetics than the non-FT one.

Table 2 compares the performance of our microfluidic devices with many recently reported MFCs with the same model biocatalyst S*. oneidensis* strain MR-1^17^,^18^,^20^,^21^,^29^,^30^,^46^,^57^. It should be noted that due to using different device structures and different cultures, it may be difficult to make point-to-point comparisons of power densities normalized to the anode surface area and the volume of anolyte chamber. Compared to recently reported devices using GF^29^,^30^, carbon cloth^20^, Au^17^,^18^,^21^, and PEDOT nanofibers^46^ as anode materials with the same *S. oneidensis* strain MR-1, the present FT μMFC exhibited a higher current density of 91.8 μA/cm^2^, a higher volume power density 745 μW/cm^3^, and a slightly lower but still competitive surface power density of 89.4 μW/cm^2^. Furthermore, compared to the large scale MFCs using graphite as an anode material^57^, our FT μMFC had a lower surface power density. However, the anolyte chambers of the previous GF- and graphite-based MFC are nearly three orders of magnitude larger than that of our device. It should be noted that the electron producing capability of strain MR-1 used in our device has limited the output current and power density. By using optimally mixed bacterial cultures, such as *Geobacteraceae*-enrichment cultures^19^,^52^, the electricity generation capability of the device could be further enhanced. Table 2 also compares the CE values of our FT with non-FT devices calculated based on the consumption of 20 mM lactate in the lactate defined minimal medium when the devices operated in batch mode (see the calculation method in Experimental section). Using the FT device as an example, *C*_T_ = *n* × *F* × *V* × [lactate] = 4 × 9.64853 × 10^4^ (C/mol) × 0.7 cm × 0.7 cm × 0.12 cm × 0.02 M = 0.454C, and *C*_P_ = 0.157 C obtained based on the integrated area in the *I*−*t* plot (not shown) in batch mode. Thus, the calculated CE of the FT device was 34.6%. As the volume of the freeway space *V*_fr_ increased from *V*_gf_ to 6*V*_gf_, the CE values of our non-FT devices decreased from 28.4% to 12.6%, further demonstrating the benefit of the microfluidic FT design.

High-throughput screening of different species of microbes (e.g., *Shewanella, Geobacteraceae, Escherichia coli*, and *Pseudomonas*) and strains is highly desirable to maximize energy harvesting through microbial conversion of organic substrates into electricity. Traditional screening methods to identify the best performing bacterial candidates are based on performing multiple experiments using large size, two-chamber MFCs, thus requiring large material consumptions and long response time due to diffusion-limited reaction and settling. As demonstrated above, our μMFCs provide high current and power densities, short start-up time within only a few hours, and reduced substrate consumptions, owing to the new porous GF-enabled FT mechanism. Furthermore, due to the use of inexpensive microfabrication techniques, it is possible to integrate multiple μMFCs in a single device to facilitate parallelization and throughput of experiments. Therefore, this μMFC technology will have a potential to realize rapid screening assays of bacterial species and strains, using electricity generation as a direct indicator for the ability of microbial power output. In addition, the μMFCs could even integrate microsystems technology to generate different growth conditions (e.g., pH, temperature, and light) for a specific bacterial strain used in all the μMFCs of an integrated device. This will allow for further screening of various environmental conditions that can influence bacterial metabolism, and thus electricity generation of strains.

## Conclusions

We have demonstrated a unique microfluidic FT μMFC with a porous GF anode sandwiched by a PEM and an electron collector at the bottom of the anolyte chamber. The built-in interconnected pores of the GF anode served as a microfluidic porous channel for flowing nutrition medium through the anolyte chamber to enhance electrochemical interactions between the colonized microbes on the scaffolds of GF and the nutrients. Mass transport of nutrients was mainly driven by pressure. In addition, molecular diffusion of nutrients to the biofilm on the scaffolds occurred directly inside the pores of the GF over a short length scale. Therefore, our FT design allowed reducing bioconvertible substrate consumption while keeping a short response time of current generation. Using *S. oneidensis* MR-1 as a model biocatalyst without any optimization of bacterial culture, the device provided 745 μW/cm^3^ volume power density based on the total volume of anolyte chamber, and 89.4 μW/cm^2^ surface power density and surface current density of 91.8 μA/cm^2^ based on the planar surface area of GF anode. The medium consumption and the current generation response time of the FT device were reduced by up to 16.4 and 4.2 times, compared to its non-FT counterpart with the freeway space volume six times the volume of GF anode.

## Additional Information

**How to cite this article**: Jiang, H. *et al*. Integrated Microfluidic Flow-Through Microbial Fuel Cells. *Sci. Rep.* **7**, 41208; doi: 10.1038/srep41208 (2017).

**Publisher's note:** Springer Nature remains neutral with regard to jurisdictional claims in published maps and institutional affiliations.

## Figures and Tables

**Figure 1:**
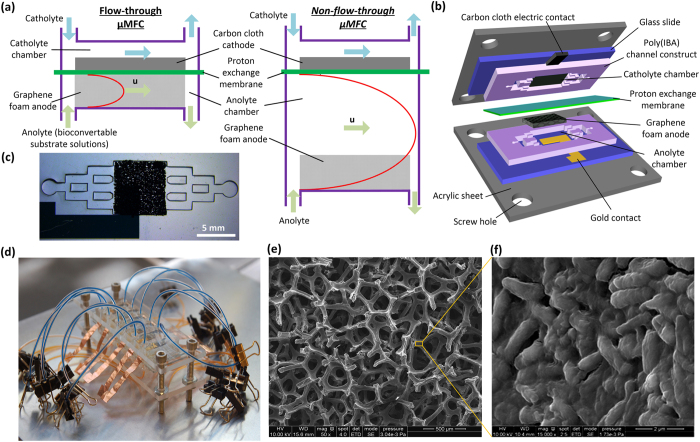
(**a**) Schematic of the proposed microfluidic FT (left) and non-FT μMFC devices (right). (**b**) Schematic of the device components for the microfluidic FT μMFC. (**c**) Optical image of the porous anolyte chamber with an embedded 3D GF anode. Diverging microfluidic channels distribute the anolyte solution flow in a relatively even manner across the width of the chamber. (**d**) Photo of a ~1 × 1 × 3 inch^3^ array of six microfluidic FT μMFCs. (**e**,**f**) Scanning electron microscopic images of the porous GF anode with a bacterial biofilm formed on the surface of the GF scaffolds. A close up view in image (**f**) shows the microbial colonization of the scaffolds in (**e**).

**Table 1:** Electrochemical parameters for the GF anodes of the FT and non-FT μMFCs using CV and EIS studies.

Culture medium	Device type	Peak current (mA)	Diffusion coefficient *D*_0_ (cm^2^·s^−1^)	Charge transfer resistance *R*_ct_ (kΩ)	Electron transfer rate constant *k*_0_ (cm·s^−1^)
TSB	FT	0.29	2.38 × 10^−10^	1.23	3.24 × 10^−11^
	Non-FT	0.08	0.20 × 10^−10^	1.44	1.94 × 10^−12^
Lactate defined minimal medium	FT	0.15	6.28 × 10^−9^	0.88	1.13 × 10^−10^
	Non-FT	0.07	1.40 × 10^−9^	2.96	2.35 × 10^−11^

**Table 2:** Performance comparison between the proposed devices and other reported μMFCs *using the same S. oneidensis strain MR-1 as a model biocatalyst.*

Anode/area (cm^2^)	Carbon cloth cathode area (cm^2^)	Anolyte chamber size(a) (μL)	Startup time(b) (hr)	Current density(c) (μA/cm^2^)	Coulombic efficiency (%)	Mean power density (Pmax)		Ref.
						Ps(d) (μW/cm^2^)	Pvc (μW/cm^3^)	
GF/0.49	0.49	58.8	5 ± 0.8	91.8 ± 4.8	34.6 ± 2.2	89.4	745	This work
GF/0.49	0.49	117.6	8.3 ± 0.6	89.6 ± 3.1	28.4 ± 1.9	88.5	368.8	
GF/0.49	0.49	176.4	9.1 ± 0.7	85.6 ± 3.2	26.1 ± 2.2	86.2	239.4	
GF/0.49	0.49	235.2	11.4 ± 0.6	88.2 ± 5.8	23.2 ± 3.1	84.8	176.7	
GF/0.49	0.49	294	12.8 ± 1.1	94.9 ± 6.2	19.8 ± 3.9	82.2	137	
GF/0.49	0.49	352.8	15.4 ± 0.8	79.8 ± 4.5	13.2 ± 3.6	85.7	119	
GF/0.49	0.49	411.6	20.8 ± 1.4	81.6 ± 4.7	12.6 ± 2.3	77.8	96.8	
GF-PANI/1	8	1.81 × 10^5^	N/A	N/A	N/A	76.8	0.42	29
GF/7~10	70	2.5 × 10^4^	N/A	71.43	N/A	96.4	27	30
Carbon cloth/0.4	0.4	4	6	10	18	0.62	62.50	20
Au/0.15	0.4	1.50	12	13	N/A	0.15	15.30	18
Au/0.38	0.38	154	N/A	1.64	N/A	0.37	0.93	17
Au/0.38	0.38	400	<13	N/A	N/A	2.35	N/A	21
Graphite plate/1.92	828	8 × 10^5^	N/A	N/A	N/A	329	0.79	57
Graphite plate/155	828	8 × 10^5^	N/A	N/A	N/A	141	27	57
PEDOT nanofibers/1	1	12	~1	16	N/A	2.54	423	46

^(a)^For our devices, the microfluidic diverging channels on the two sides of the porous anolyte chamber are excluded in the volume calculation.

^(b)^Data for our devices are obtained from the black curve (at TSB medium flow rate of 20 μL/hr) in Fig. 6b.

^(c)^The current values of our devices refer to 80% of the maximum current shown in Fig. 6a.

^(d)^The mean values of surface power density for our devices are calculated based on the planar surface area of the anode electrode.
